# Hiring a team: an integral selection procedure for inter- and transdisciplinary PhD candidates

**DOI:** 10.1057/s41599-026-08028-8

**Published:** 2026-07-07

**Authors:** Annemarie Horn, Noelle M. N. C. Aarts, Irma Arts, Joris P. G. M. Cromsigt, Martin A. M. Drenthen, Sabrina Dressel, Gerard Schouten, Svenja Waldmann, Ine Dorresteijn

**Affiliations:** 1 Liberal Arts and Sciences, Philophy & Religious Studies, Utrecht University, Utrecht, Netherlands; 2 Institute for Science in Society, Radboud University Nijmegen, Nijmegen, Netherlands; 3 Department of Wildlife, Fish and Environmental Studies, Swedish University of Agricultural Sciences, Umeå, Sweden; 4 Copernicus Institute of Sustainable Development, Utrecht University, Utrecht, Netherlands; 5 Centre for African Conservation Ecology, Nelson Mandela University, Gqeberha, South Africa; 6 Forest and Nature Conservation Policy Group, Wageningen University & Research, Wageningen, Netherlands; 7 Fontys University of Applied Sciences, Eindhoven, Netherlands

**Keywords:** Psychology, Psychology, Science, technology and society

## Abstract

In the inter- and transdisciplinary (ITD) research project WildlifeNL, we hired six PhD candidates through a selection process that explicitly valued ITD competencies and aligned the process to hire a balanced team of PhD candidates. The approach included (1) a rubric with individual and team assessment criteria that we used across positions; (2) selection procedures that were aligned in their timing; (3) alignment meetings to discuss candidates and approaches across positions; and (4) overlapping supervision teams and selection committees. In this Comment, we describe our approach and share our experiences in implementing it. We argue that designing an explicit and intentional process to safeguard the ITD character of a project in PhD hiring procedures helps to make well-grounded choices that shape the individual PhD candidates’ experiences as well as the overall ITD project and thereby supports ITD research in which PhD candidates play an important role. We experienced the rubric to be particularly valuable to mediate negotiation of expectations, values and assumptions across supervisors and selection committees. Moreover, we argue that ITD research requires not only considering the merits of individual candidates, but also their fit with the project, the consortium, and with other candidates. We argue that the central role that PhD research plays in many ITD research contexts necessitates intentional hiring which includes explicit assessment criteria for team composition, diversity, and ITD competencies. We invite others to translate our experiences in ITD PhD hiring processes to their context, including the rubric that we make available in this article.

## Need for inter- and transdisciplinary hiring

Inter- and transdisciplinary (ITD)^1^ research is commonly adopted to make research more responsive to societal issues (Jacobi et al., 2022) and funding schemes increasingly ask for cross-disciplinary collaboration and societal engagement (Ramos-Vielba et al., 2022). Consequently, PhD candidates also increasingly conduct their research in ITD contexts. Both research studying ITD PhD candidates (e.g., Bulten et al., 2021; Felt, 2014; Haider et al., 2018) and reflective self-reports by ITD PhD candidates (Bridle et al., 2013; Djinlev et al., 2023; Rogga & Zscheischler, 2021) paint a picture of the challenges that ITD research brings. These challenges include the demand for different and additional competencies compared to more disciplinary research (Guimarães et al., 2019), combining conflicting roles (Bulten et al., 2021; Schuijer et al., 2021; Wittmayer & Schäpke, 2014), and a scattered sense of belonging (Felt, 2014). PhD candidates are often insufficiently prepared for the tasks and roles that they are responsible for, which can lead to high emotional costs (Enright & Facer, 2017).

The performance of PhD candidates is important for the success of many research projects. They often conduct much of the research, such as collecting and analyzing data. This also holds for ITD research projects, implying that success of ITD research strongly relies on the success of the ITD research conducted by PhD candidates (Eschen et al., 2025; Horn & Krabbenborg, 2025). Therefore, supporting PhD candidates in the abovementioned different roles and competencies serves not only their own learning process, but also project success. This calls for training, support and supervision of ITD PhD candidates (Eschen et al., 2025; Kovacic & Marcos-Valls, 2023), but also for selection of PhD candidates with profiles and competencies that equip them for this role (Stoof et al., 2020).

This motivated us to design a selection and hiring procedure focused on ITD competencies and motivations when hiring six PhD candidates for the ITD project WildlifeNL^2^. The core of the research in WildlifeNL is done by a group of PhD candidates in close collaboration with each other, societal partners, and their PhD supervision teams, conducting research with different (disciplinary) research foci: ecology (2 PhD candidates), human dimensions, philosophy, communication science, and governance. Here, we present our hiring approach, which intentionally assessed ITD competencies at individual and group level. We argue that intentional assessment of team composition, diversity, and ITD competencies will strengthen the key role that PhD research plays in many ITD projects. We hope to inspire others in shaping their own ITD hiring processes building on the concrete and practical tools that we provide in this Comment.

## The WildlifeNL selection and hiring approach

### Joint negotiation of expectations and values

We negotiated the competencies and assessment criteria among all PhD supervisors. The first author of this article (AH) joined the WildlifeNL project supporting and studying the ITD research process, as an integration expert (Hoffmann et al., 2022) and accompanying researcher (Christensen et al., 2016). In that role, she conducts auto- and collective ethnographic research on the ITD research processes in the project (Lapadat, 2017).^3^

In June 2024, AH facilitated an open conversation in which she collected key criteria that the Principal Investigators (PIs: ID, JC, MD, GS) of the project found important in the PhD candidates on the project. This conversation was informed by AH’s knowledge about the literature on ITD competencies and challenges. This conversation covered the topic of responsibility for knowledge integration in the project, based on reports that knowledge integration can unevenly land on the shoulders of early career researchers (Djinlev et al., 2023; Rogga & Zscheischler, 2021) or remain underconsidered when ownership is not explicitly allocated (Hoffmann et al., 2022). Finally, insights about competencies for ITD research, such as openness to other perspectives and ability to contribute own expertise (Horn et al., 2022) were included in this discussion. Moreover, the conversation tapped into the prior experiences of ITD research and supervision among the researchers present in the meeting.

**Fig. 1:**
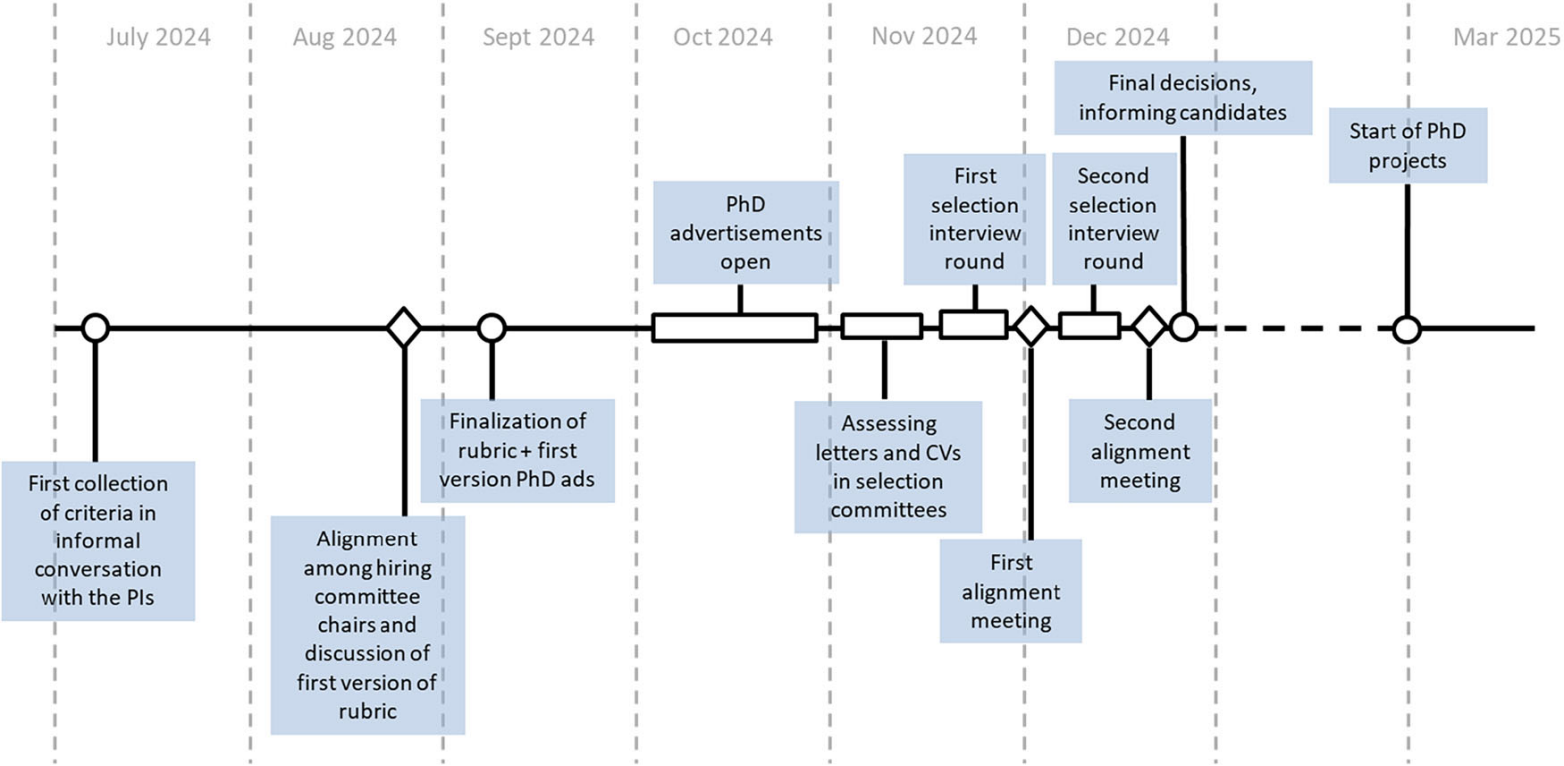
Timeline of the selection procedure between July 2024 and start of the PhD candidates in March 2025. Single timepoint events are indicated with a circle or diamond, whereas events that lasted a period are indicated by rectangles. The key moments for alignment are signified with diamond shaped markers. Events that are internal are positioned on the lower part of the figure, below the timeline, whereas external events that included communication toward the potential PhD candidates are indicated above.

AH compiled the priorities articulated by the future supervisors into a selection rubric based on the work by Stoof et al. (2020) and circulated this draft to all main supervisors for the PhD positions, who also chaired the selection committees. They provided input by email and discussed the rubric in a meeting in August 2024. AH finalized the rubric based on this meeting. In this meeting, they also made decisions on the composition of the selection committees and timing of the main steps in the hiring procedures. The timeline is displayed in Fig. 1 and the resulting rubric in Table 1.

**Table 1:** Rubric for assessment of candidates based on letters, CVs and selection interviews in hiring inter- and transdisciplinary PhD candidates for the WildlifeNL project.

		Absent	Present	Outstanding
1. Scientific (disciplinary) potential	1a. Relevance of degree	[defined per PhD position, indicating the range of degrees that were considered appropriate, research methods that were considered key, and discipline-specific skills]		
	1b. Demonstrated research capacities			
	1c. Demonstrated experience with academic writing/ demonstrated capacity in academic writing			
2. Communication & teamwork (inter- and transdisciplinarity)	2a. Ability to effectively communicate to diverse audiences (different disciplines, different actors)	Does not demonstrate the ability to translate specialized knowledge (e.g. from master’s thesis) to a broader audience; does not demonstrate awareness of importance of communicating with diverse academic and non-academic audiences.	Demonstrates awareness of importance and willingness to engage in communication with diverse academic and non-academic audiences.	Demonstrates the ability to convey specialized knowledge from their study background to a non-expert audience. Additional merit: experience with inter- and/or transdisciplinary research.
	2b. Stakeholder engagement	Does not demonstrate awareness of diversity of consortium and the implications of working and communicating with diverse actors.	Demonstrates awareness of diversity of consortium and implications for collaborating, interacting, and communicating with them; expresses enthusiasm about working in a diverse consortium and engaging with diverse partners.	Demonstrates sensitivity to diversity of actors and different ways of addressing and reaching them. Additional merit: demonstrated experience with stakeholder engagement in research or other professional activities.
	2c. Societally relevant and/or applied research	Primarily interested in fundamental research and does not express intent to generate societally robust or practice-oriented insights and outputs.	Expresses intent to generate insights with value for practice and societal robustness.	Provides concrete insights into how they intend to ensure practical relevance and societal robustness of research findings. Additional merit: demonstrated relevant experience.
	2d. Open-mindedness; being able and willing to see things from different perspectives	Speaks and/or writes exclusively from a single perspective (e.g. academic or urban).	Expresses the importance of being humble, suspending judgment, listening well in writing/ speaking *about* open-mindedness.	Demonstrates ability to listen well and actively, takes a humble stance in relation to the limitations of their own knowledge and/or novel information that is shared with them.
	2e. Teamwork and collaboration in research	Indicates to prefer to work alone and/or to not experience added value of working in a team.	Demonstrates experience with teamwork and reflects on its yields.	Expresses concrete advantages and challenges of collaborating in a team and reflects on own behaviours and preferences in teamwork.
3. Motivation & commitment	3a. Motivated interest in research topic of PhD, approaches and methods	Motivation remains unspecific to the research topic of the PhD without details about relevant methods, theories and approaches.	Refers to specific aspects of the research approach of the position that appeal to them; e.g. methods, theories, topics.	Has concrete ideas of how to shape the research within the frames of the PhD project that align with the scope and intentions of the WildlifeNL project and expertise and interests of the supervision team.
	3b. Motivated interest in the topic of human-wildlife interactions	Motivation remains unspecific to the content of the WildlifeNL project; candidate shows to be poorly informed about the project.	Refers to specific aspects of the project that appeal to them; is informed about the project based on publicly available information.	Has personal and/or professional experience with topics related to human-wildlife interactions that their interest is grounded in.
	3c. Motivated interest in working inter- and transdisciplinarily	Inter- and/or transdisciplinary nature of the project remains unmentioned or unspecific in the motivation expressed.	Expresses explicit enthusiasm about working in an inter-and/or transdisciplinary manner.	Provides concrete pointers of what about inter- and/or transdisciplinary research appeals to them.
	3d. Willingness to spend (prolonged periods of) time in the field	Expresses to be unable and/or unwilling to spend (prolonged periods of) time in one or both of the living labs, otherwise in the field, or at the university locations at which they will be based.	Is willing to spend time in the field and in the office, framing this as “necessary”.	Expresses explicit enthusiasm about spending time on location and is explicit about what that may bring them.

### Aligned selection processes

We aligned the selection and hiring process in terms of both content and timing. This enabled the selection procedures for the individual PhD positions to inform each other and thereby allow decision-making at the team level, aiming for complementarity and fit at the PhD team level. We published all six PhD advertisements simultaneously on the first of October 2024, and the selection committees agreed on fixed weeks to select shortlisted candidates based on their motivation letters and CVs, and for the first and second rounds of selection interviews. This allowed us to align between selection committees around all key decision moments.

### A shared assessment rubric

The rubric we developed served as a starting point for formulating the PhD advertisements, for assessing the candidates based on their letters and the selection interviews, and for directing conversation in the alignment meetings among the chairs of the committees.

The rubric consisted of two parts: assessment criteria for the individual candidates (Table 1) and a list of requirements and merits at the team level (Box 1).

The rubric was divided into three main categories: (1) position-specific criteria formulated for each position by the selection committee, which included relevance of degree (1a), research experiences and skills (1b), and academic writing (1c); (2) collaboration and communication; and (3) motivation.

Besides the rubric used for individual assessments, we also formulated a list of criteria that were seen as necessary and/or desirable at the team level (Box 1). These were not used to assess candidates individually but served as a checklist to ensure that important assets were present at the team level and to structure conversations about team composition during the alignment meetings. We implemented the diversity criteria loosely as discussion points, as we found it important to avoid essentializing differences or categorizations. For instance, we talked about feminine and masculine qualities rather than a binary gender qualifications.

Box 1 Team level criteria Minimum team requirements:

**Table:** 

	Minimum number of PhD candidates in the team
Fluent in Dutch language	4
Experience with qualitative data collection and analysis	1
Experience with ArcGIS	1
Experience with statistical modeling	2
Experience with field experiments	1
Experience with field observations	1
International wildlife research experience	1
Wildlife research experience in Dutch context	1
Drivers license	2

Diversity criteria:

- Geographical and cultural diversity:
  ○ From different Dutch—and possibly Belgian—provinces
  ○ Representing not solely urban perspectives
  ○ Candidates with non-Dutch backgrounds and experiences as additional merit (given its commensurability with language fluency requirements)
- Gender diversity

### Alignment of selection interviewing approach

Besides the above-described timing, we also aligned the approaches we took for the selection interviews with candidates. We discussed the interviewing approach, including potential questions, in the first alignment meeting when all six PhD selection committees met (Nov 2024). For the first interview round, all committees followed a similar structure and included nearly identical questions. During the alignment meeting, several points from the rubric surfaced which were challenging to assess. For instance, we discussed how to assess open-mindedness. We agreed that each committee would include the same real-life example from the project in which perspectives between hunters and terrain managers clashed. We asked all candidates how they would deal with the issue to assess their sensitivity to the topic, and whether they would stay open-minded and curious despite potentially being personally biased towards one perspective. Moreover, we agreed that each committee would ask for specific examples about teamwork, personal experiences, and motivation for doing PhD ITD research.

### Composition of supervision teams and selection committees

A last feature of the approach we took to hiring a team of PhD candidates was the composition of the selection committees. Based on the original research proposal and focus of the PhD projects as described in the WildlifeNL research agenda (Arts et al., 2026), we composed supervision teams of three or four supervisors from different disciplines and higher education institutions. There was substantial overlap between the supervision teams. In total, 11 different supervisors were involved, each co-supervising between one and four PhD candidates. The selection committees for the hiring procedure closely followed the supervision teams but also included PhD candidates from the participating institutions. This meant that the selection procedure represented the same diversity as the supervision teams.

## Experiences and reflections

Now, how did this approach unfold? How did it affect the selection process and the candidates ultimately hired? What were some of the main challenges?

At the time of writing this article, six PhD candidates were hired and were about half a year into their research. This allows us to look back at the hiring procedure, the team we hired and their onboarding. Below, we provide some lessons and considerations based on our experiences. To illustrate experiences we use quotes from alignment meetings and interviews with the author team done by an independent researcher as part of a related research project.

### How the hiring approach shaped the team we hired

The key question arises whether our intentional selection approach caused us to hire different candidates. While we cannot make a comparison, we do have some indications of how the selection committees approached the hiring procedure differently because of its ITD nature. Supervisors repeatedly reflected that candidates who may have been excellent for other—more disciplinary or more solitary—positions were deemed less fitting for the PhD positions in this project. A supervisor said the following about a candidate during one of the alignment meetings: “*This candidate is more theoretical, very good, but not for a transdisciplinary project. [..] from a [disciplinary] perspective they would be best for me to work with, but the risk [for the project] is too big”* (second alignment meeting, Dec 2024). Another committee member explained how the rubric enabled them to value other competencies and pulled them away from more disciplinary assessment of candidates:

*“there was a candidate who was analytically very strong and already had publications and a track record. So, if we had looked at the standard, classic way of rating them, that person would have been number one. But then when you think about their answers about conflict resolution, communication and more soft skills and thinking also that that person needs to connect with very diverse topics in how they think about interdisciplinarity… It [the rubric] gave arguments for the gut feeling that this person did not fit the project.”*

As a result, we argue that the ITD nature of the project shaped the selection decisions, valuing different assets compared to when hiring for more disciplinary PhD positions.

### Nurturing dialogue about values and assumptions

The way the different selection committees used the assessment rubric differed substantially. In some committees, each member used it explicitly to score each candidate; then collating the independent scores from all committee members to create a shortlist. Other committees followed the rubric more loosely as a reminder of the qualities that we agreed to consider. Personal, disciplinary, and institutional differences played a role shaping these different uses.

What the committees collectively perceived as most valuable about the rubric, however, was that it served as a boundary object to discuss expectations, values and assumptions about the PhD positions upfront, and to guide discussions among selection committees about ranking of candidates. As one committee member put it:

“*Maybe it does not per se matter that it took the form of a rubric, but rather the fact that we used the rubric to engage in a systematic and thorough discussion, to have a conversation about what kind of candidates we were looking for. […] and not only the skills or expertise for the project, but also other traits and diversity of the team. […] so, we used the rubric to formalize this.”*

### From individual to team excellence

We approached the hiring procedures as the collective hiring of a team of six PhD candidates by describing the team level criteria (Box 1) upfront and discussing them in the alignment meetings. One member described how the team criteria and alignment worked out in practice and what it yielded for the hiring procedure:

“*Imagine having six candidates, one of whom is very good but has little affinity with stakeholder engagement, while some of the other five do. Then the question becomes, can this be compensated in the team? So then it becomes less important that one candidate has it all, but instead you can look at the team level: with the candidates we have in mind, do we have enough experience with stakeholder engagement on board? […] So, when you hire a team, it can help to make sure that all necessary competencies are represented in the team”*

This illustrates that committees experienced the team level criteria and alignment as a workaround for looking for a ‘unicorn’ or ‘five-legged sheep’. Instead of having to find that one candidate who ticks all the boxes, this allowed flexibility to look for complementary candidates.

Overlapping supervision teams and hiring committees aided alignment. Besides more structured and formal alignment in the alignment meetings (see Fig. 1), it also happened naturally through the supervisors who were part of multiple committees and were automatically informed about multiple hiring processes, as illustrated by the following experience:

“*Two colleagues in our committee were also in two or three others. They also gave updates, saying that they were circling in on people that were really strong and have this or that, which gave us a bit of leeway maybe selecting someone we really liked but who doesn’t have that specific asset*.”

### Practical challenges: workload and institutional logistics

Although our approach led to hiring a promising team of PhD candidates, the approach also brought several challenges: it was labor intensive and logistically challenging.

Extra work went into drafting and negotiating the assessment rubric and the alignment meetings prior to and during the candidate selection and interviewing stages. The negotiation of the assessment rubric took two meetings (see Fig. 1) and included feedback and input on different version of the rubrics and assessment criteria, and extra work from AH as dedicated integration expert. However, we experienced the amount of work as manageable, and its value outweighed the additional work. We especially experienced the process of explicating and negotiating expectations, values, and assumptions as valuable for the ITD hiring process.

Including two different universities in the selection process added another challenge because each had different hiring practices and policies. These differences included: maximum length of PhD advertisements, maximum number of qualities listed in the advertisement, institutional policies around timing of advertising and informing, and the possibility to post multiple advertisements on a single page. These differences meant that we had to deviate from our ideal of one big “clustered” advertisement for all six positions. Instead, we published them as separate postings on the university websites with each posting referring to the others, and a single clustered advertisement on the project website. For other, larger and international projects, with larger numbers of institutes involved and potentially larger diversity in national and institutional conventions and policies, this is likely even further complicated.

Lastly, we acknowledge that a team hiring approach does not ensure teamwork once the PhD candidates have started their research. We want to emphasize that hiring may support and put conditions in place, but that being and becoming a team also requires continuous investment in onboarding, team-building, supervision, and training. Therefore, these are things that we also explicitly invest in in the WildlifeNL project, among other things, through weekly meetings and regular training activities for the project’s Early Career Researchers team.

## Recommendations for hiring PhD candidates in ITD research

We argue that designing explicit and intentional processes for hiring PhD candidates is important for ITD. Organizing alignment prior to selection helps negotiate and align assessment criteria for PhD advertisement, letters, and selection interviews. A rubric can mediate this process. Moreover, we argue that ITD research requires not only considering the merits of individual candidates, but also their fit with the project and with other candidates. Furthermore, we experienced the use of a case from an ITD research project in the selection interview as a fruitful way to gain insight into the candidates’ attitudes and sensitivity towards diverse (actor) perspectives. Lastly, we experienced the disciplinarily diverse and overlapping supervision teams as key success factors for an integral hiring approach, as well as for shielding the PhD candidates from becoming (overly) responsible for ITD integration activities.

Rather than providing a universal blueprint for hiring ITD PhD candidates, we aim to inspire others and to contribute to a research culture in which we share our experiences to collectively learn. We encourage others to use our lessons, criteria and rubric and translate them to their ITD context.

## Data Availability

This manuscript does not report data generation or analysis.

## Footnotes

1 In this article, we consider inter- and transdisciplinarity as a single term, not intending to disregard their specificities and heterogeneity both across and within these categories, but rather because we share our experiences from a context that is characterized by a combination of inter- *and* transdisciplinarity. Exactly the combination of collaboration between diverse academics and with diverse practice partners is integral to the ITD approach of WildlifeNL. We believe that some but not all lessons can be linked exclusively to either the inter- or the transdisciplinary nature of this project and aim to inspire others whose practices also combine both modes of collaboration.

2 WildlifeNL is funded by the National Science Agenda program of the Dutch Research Council, NWO. WildlifeNL brings together a consortium of five knowledge institutions and 15 societal partners to investigate ways to facilitate the coexistence of humans and large mammals in the Netherlands. For more information about the content and approach of the project, we direct readers to the website of the project: www.wildlifenl.nl. More information about the ITD design of the project can be found in Arts et al. (2026).

3 See Arts et al. (2026) for a more detailed description of this meta-research on ITD research in the WildlifeNL project.
